# Bioprinting of a multi-composition array to mimic intra-tumor heterogeneity of glioblastoma for drug evaluation

**DOI:** 10.1038/s41378-024-00843-w

**Published:** 2024-12-11

**Authors:** Gihyun Lee, Soo Jee Kim, Yejin Choi, Jongho Park, Je-Kyun Park

**Affiliations:** 1 Department of Bio and Brain Engineering, Korea Advanced Institute of Science and Technology (KAIST), 291 Daehak-ro, Yuseong-gu, Daejeon 34141 Republic of Korea; 2 KI for Health Science and Technology, KAIST Institutes (KI), 291 Daehak-ro, Yuseong-gu, Daejeon 34141 Republic of Korea; 3 KI for NanoCentury, KAIST Institutes (KI), 291 Daehak-ro, Yuseong-gu, Daejeon 34141 Republic of Korea

**Keywords:** Organic-inorganic nanostructures, Nanofabrication and nanopatterning

## Abstract

Microextrusion printing is widely used to precisely manufacture microdevices, microphysiological systems, and biological constructs that feature micropatterns and microstructures consisting of various materials. This method is particularly useful for creating biological models that recapitulate in vivo-like cellular microenvironments. Although there is a recent demand for high-throughput data from a single in vitro system, it remains challenging to fabricate multiple models with a small volume of bioinks in a stable and precise manner due to the spreading and evaporation issues of the extruded hydrogel. As printing time increases, the extruded bioink spreads and evaporates, leading to technical problems that decrease printing resolution and stability, as well as biological problems that affect 3D culture space and cell viability. In this study, we describe a novel microextrusion bioprinting technique to stably fabricate a multi-composition array consisting of massive and nanoliter-scale hydrogel dots by using multi-bioink printing and aerosol-based crosslinking techniques to prevent spreading and evaporation issues. We confirmed that the crosslinking aerosol effectively prevented spreading and evaporation by analyzing the morphological changes of the extruded hydrogel. By adjusting the extruding ratio of the bioinks, we were able to print a multi-composition array. This stable and massive array printing technique allowed us to improve the replicates of biological models and provide various data from a single culture system. The array printing technique was applied to recapitulate the intra-tumor heterogeneity of glioblastoma and assess temozolomide efficacy on the array model.

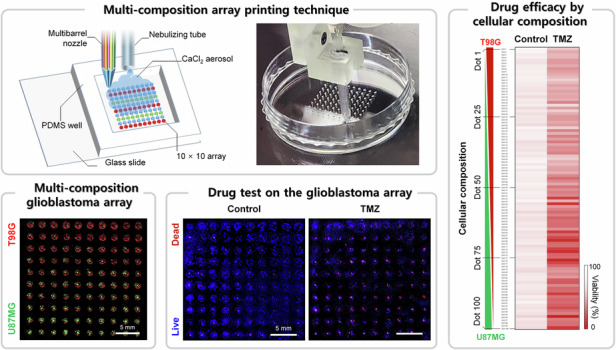

## Introduction

Microextrusion printing, a manufacturing technique to precisely fabricate micropatterns and microstructures with various materials^1–3^, has commonly been applied in many research fields providing microdevices^4–8^, microphysiological systems^9–11^, and biological constructs^12–15^. A general microextrusion printing system is equipped with one printing nozzle per printhead, allowing it to dispense specific ink following a programmed trajectory. By using more printheads^12,16–18^ or special nozzles^15,19–22^, the printing system can extrude various materials to fabricate complex models with multiple compositions. In biofabrication and tissue engineering fields, existing printing technologies have been shown to construct more complex in vitro models for cell culture and analysis with cell-containing hydrogel bioinks^23–26^. Although these printing techniques could recapitulate complex cellular microenvironments, a strategy to improve data throughput from a single model is necessary^27^.

Microextrusion bioprinting can not only mimic specific tissues or organs^15,23,28,29^ but also provide high-throughput data by improving replicates on a single model^27,30–33^. If multiple bioink printing technology is utilized, data throughput can be improved by implementing various experimental conditions in one array model^33,34^. However, the stable fabrication of multiple in vitro models with a small volume of bioinks remains challenging due to the spreading and evaporation issues of the extruded hydrogel. As the printing time increases, a small volume of the extruded bioink gradually spreads to the surface of the substrate, and water molecules of the hydrogel evaporate. Spreading reduces printing resolution, which limits the ability to precisely fabricate the desired geometries^26,28^. Evaporation causes a loss of hydrogel volume, leading to a decrease in cell viability^18^. For these reasons, the spreading and evaporation issues that occur during the printing process make it difficult to massively produce in vitro models consisting of small volumes of bioink. The spreading and evaporation issues could be minimized by decreasing the temperature of the printing substrate or modifying the surface of the substrate^35^. However, these methods would affect cell viability and require additional complicated processes to fabricate in vitro models. Furthermore, existing microextrusion printing systems based on multiple printheads to create in vitro models of various compositions in one array require a long printing time, including the nozzle replacement process, making precise manufacturing difficult.

In this study, we describe a massive and stable bioprinting technique of nanoliter-scale hydrogel dots and apply it to fabricate a multi-composition glioblastoma (GBM) array representing intra-tumor heterogeneity of the brain tumor for drug efficacy tests. To fabricate the small volume and massive hydrogel dots, we select an aerosol-based crosslinking approach that can provide a humid environment as well as gelate the extruded materials during the printing process. The crosslinking aerosol improves printing stability, resolution, and production yield for the hydrogel dots. With the massive and stable bioprinting technique, we fabricate a multi-composition GBM array to mimic the intra-tumor heterogeneity of brain tumors by using a multibarrel nozzle that can continuously extrude multiple bioinks. The multi-composition GBM array consisting of 100 different configurations is used to analyze the effect of intra-tumor heterogeneity on drug efficacy tests.

## Results and discussion

### Improvement of printing stability and resolution of nanoliter-scale hydrogel dots

We established a stable fabrication technique of hydrogel dots with high resolution using microextrusion bioprinting that can effectively meet the needs of in vitro humanized modeling through easy manipulation, precise positioning, and direct extrusion of a wide range of bioinks^36,37^. However, we encountered challenges because the bioink extruded from the nozzle was immediately exposed to the external environment, leading to spreading and evaporation during the printing process. From a technical perspective, the spreading of the extruded bioink reduces the printing resolution^15^. In particular, fine patterns of a small volume and microstructures that require a prolonged printing process were susceptible to evaporation. Evaporation of the bioinks is a major cause of an irregular change in material composition and a significant reduction in cell viability^35^. The aerosol-based crosslinking technique integrated into the multi-bioink printing system is beneficial in preventing technical and biological issues such as spreading and evaporation^15,38^.

**Fig. 1:**
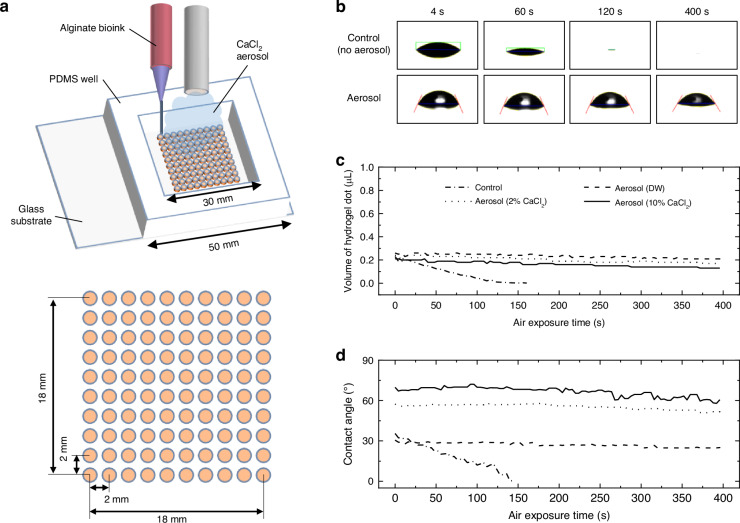
Concept of stable microextrusion printing technique using crosslinking aerosol to fabricate a small-volume hydrogel array. **a** Schematic for printing a 10 × 10 hydrogel dot array with a multibarrel nozzle and crosslinking aerosol. **b** Time-lapse images of the extruded 2% alginate dot exposed to air and 10% CaCl_2_ aerosol for 400 s. **c** Change in volume of the hydrogel dot exposed under the various crosslinking conditions for 400 s. **d** Contact angles of the extruded hydrogel dot to analyze the effect of the crosslinking aerosol during the printing process

To confirm the printing stability and applicability of a small volume of bioink, a 10 × 10 array model consisting of 100 hydrogel dots spaced 2 mm apart was designed, and alginate ink was printed without or with CaCl_2_ aerosol in a polydimethylsiloxane (PDMS) well bonded on a glass slide, as shown in Fig. 1a. Before printing the array model, we first analyzed the effect of the aerosol on a single alginate dot. When the extruded alginate dot was exposed to air, the hydrogel dot rapidly dried (Fig. 1b). On the other hand, the aerosol was able to maintain the shape of the hydrogel dot during the prolonged printing process for about 400 s. Although the ~250 nL hydrogel dot was completely dried at 120 s after extruding, the aerosol groups could maintain the volume of the hydrogel dot when supplying the distilled water (DW), 2% CaCl_2_, and 10% CaCl_2_ aerosol (Fig. 1c). The aerosol composition did not affect the maintenance of the hydrogel volume, but the contact angle exhibited significant changes. Extruding alginate ink under the crosslinking aerosol influenced the spreading on the substrate (Fig. 1d). Higher concentration of CaCl_2_ aerosol resulted in a higher contact angle due to the faster gelation of the alginate dot. In the aerosol groups, the contact angle of the extruded alginate dot was maintained for 400 s. Among the three crosslinking aerosol conditions, the 10% CaCl_2_ aerosol exhibited the most stable and highest hydrogel dot, indicating that it could prevent the hydrogel from spreading on the substrate and retain the hydrogel height. Consequently, we verified that the crosslinking aerosol supplied in the printing process improved printing stability and resolution.

### Microextrusion printing of a nanoliter-scale hydrogel dot array

**Fig. 2:**
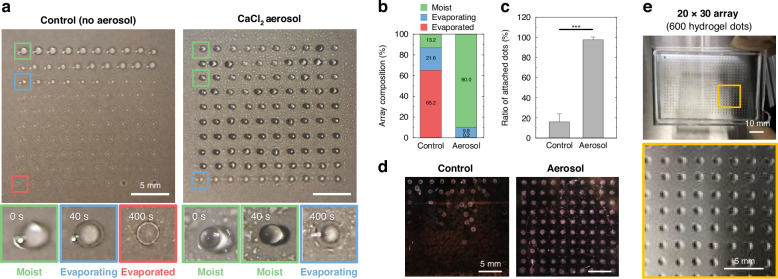
Establishment of stable printing technique of a small-volume and massive hydrogel array. **a** 10 × 10 alginate dot array printed for 400 s without or with 10% CaCl_2_ aerosol. The moist, evaporating, or evaporated hydrogel dot images captured at the various air exposure times after extruding. **b** A quantitative comparison of the composition in the whole array printed without or with the aerosol (*n* = 5). **c** The ratio of hydrogel dots attached to the substrate in the control group and nebulizing group (*n* = 5). **d** The whole array of images showing the effect of crosslinking aerosol to improve the adhesion of the small volume of hydrogel dots. **e** The massively printed array with 600 hydrogel dots using the bioprinter with the crosslinking aerosol. ***p < 0.001

After analyzing the effect of crosslinking aerosol, we confirmed whether the 10% CaCl_2_ aerosol could affect the fabrication of the nanoliter-scale hydrogel array. A 10 × 10 array consisting of 250 nL alginate dots was printed for 400 s, both without or with the crosslinking aerosol (Fig. 2a). The hydrogel array of the control group without the aerosol showed a tendency to evaporate sequentially, starting with the hydrogel dot extruded first. In the magnified images, the moist bioink exposed to an external environment during the printing process rapidly evaporated before finishing the printing process. Since the fully dried hydrogel cannot provide a suitable 3D cell culture environment, almost all the hydrogel dots of the control group cannot be used as an in vitro model. On the other hand, almost all the alginate dots of the 10% CaCl_2_ aerosol group maintained a dome shape. Even though the first extruded hydrogel dot was exposed to an external environment for 400 s, it was not completely dried by the aerosol. While the 10 × 10 hydrogel array printed without aerosol was more than half completely dried, only less than 1% of the hydrogel array in the aerosol group was fully dried after 400 s of printing (Fig. 2b). In addition, as the air exposure period was longer, the evaporation of the hydrogel dots was faster, and the dot volume was rapidly decreased in the control group (Fig. S1a). However, the crosslinking aerosol was able to improve the uniformity of the dot volume in the array. The 10 × 10 array in the control group could not maintain the volume homogeneously until the end of the printing process due to spreading and evaporation (Fig. S1b). On the other hand, the aerosol group exhibited larger and more homogeneous volumes of the dots in the array.

To fully gelate inside the alginate, the hydrogel array printed under the aerosol was immersed into the 2% CaCl_2_ solution for 5 min. After the second crosslinking step, we were able to observe how the crosslinking aerosol affected the adherence of the hydrogel dots on a substrate. The ratio of the hydrogel dots attached to the substrate was lower than 20% in the control group (Fig. 2c). However, when supplying the 10% CaCl_2_ aerosol during the printing process, approximately 97% hydrogel dots remained on the substrate. We confirmed that the dried hydrogel dots easily detached from the substrate, and that the crosslinking aerosol enhanced the stability of the array model fabrication (Fig. 2d). Since the crosslinking aerosol was able to prevent the evaporation issue, we could observe that the hydrogel array printed under the 10% CaCl_2_ aerosol adhered to the substrate. To assess the possibility of improving model replicates and throughput based on the stable array printing technique, we tried to massively fabricate nanoliter-scale hydrogel dots within 400 s. We were able to produce the maximum 600 hydrogel dots in a 20 × 30 array without sample loss for 400 s (1.5 hydrogel dots per second), as shown in Fig. 2e. The array production yield of 600 hydrogel dots was 99.83 ± 0.17%. The microextrusion array printing technique with an aerosol-based crosslinking approach enabled the stable fabrication of the massive hydrogel dots, minimizing evaporation and spreading, even though the hydrogel dots are nanoliter-scale.

### Printing of a multi-compositional array with heterogeneous patterns

**Fig. 3:**
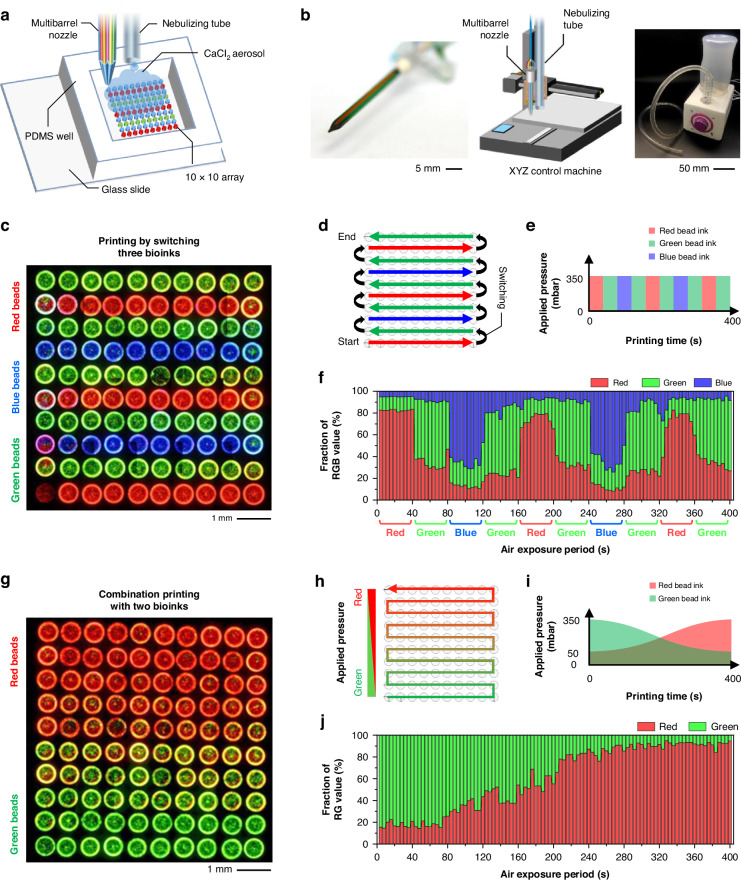
Printing of the variously patterned multi-composition arrays using the multibarrel nozzle and crosslinking aerosol. **a** Schematic of bioprinting of multi-composition hydrogel array. **b** A bioprinting system installed with a multibarrel nozzle and a nebulizer. **c** The straight pattern array printed via switching of three different bead–hydrogel inks (*n* = 3). **d** A trajectory to move the printhead for the straight pattern array. **e** Pneumatic pressure to supply through each barrel of the nozzle for the straight pattern array. **f** The fraction of red (R), green (G), and blue (B) values measured from the 100 hydrogel dots of the bioprinted straight pattern array. **g** The gradient pattern array printed by combining the two different bead–hydrogel inks. **h** A trajectory to move the printhead for the gradient pattern array. **i** Pneumatic pressure to supply through each barrel of the nozzle for the gradient pattern array. **j** The fraction of red and green values measured from the 100 hydrogel dots of the bioprinted gradient pattern array

To fabricate a heterogeneously patterned array consisting of nanoliter-scaled and multi-compositional hydrogel dots, the multiple-bioink printing system was used, as shown in Fig. 3a. The multiple-bioink printing system was configured to continuously extrude various materials through a multibarrel nozzle while simultaneously supplying aerosol via a nebulizing tube, following the previous setup protocol (Fig. 3b)^22^. The multibarrel nozzle can sequentially exchange or combine up to seven bioinks by adjusting the pneumatic pressure applied to each bioink during the printing process. During printing, a crosslinking aerosol generated from a nebulizer was supplied to the extruded bioink through a tube, which simultaneously led to the gelation of the hydrogel.

With this continuous multi-material printing system, we fabricated multi-compositional arrays that have various heterogeneous patterns. A 10 × 10 array with different compositions in each row was fabricated by extruding three bioinks, including 10 μm green, red, and blue fluorescent beads (Fig. 3c). In the strip-patterned array, each bioink was extruded along a programmed trajectory, switching to another bioink at the end of each row (Fig. 3d). At the material exchanging point, the applied pneumatic pressure channel was switched to another channel to sequentially extrude the next bioink, as shown in Fig. 3e. The composition of each hydrogel dot in the striped array was analyzed with a fraction of the RGB values from the fluorescence images. We confirmed that each fluorescence bead ink was sequentially printed row-by-row according to the pressure sequence (Fig. 3f). Furthermore, the multi-material printing system enabled a combination printing with two bioinks, including the 10 μm red and green fluorescent beads (Fig. 3g). By gradually adjusting the combination ratio of the two inks following the printing trajectory (Fig. 3h, i), a multi-compositional array with a gradient pattern could be fabricated. The pixel values of red and green of 100 hydrogel dots were consistent with the applied pressure profile (Fig. 3j). Based on the multi-material printing technique, we were able to fabricate the nanoliter-scale multi-compositional hydrogel array, which can improve the number of model replicates and provide various types of data on a single array model. Although the materials in each hydrogel dot were not homogeneously mixed in the current printing system, this problem could be solved if a rotational multimaterial printing technique was applied in our printing system. Recently, Jennifer Lewis’s group developed the rotational printing nozzle, which can not only extrude multiple materials but also rotate the nozzle for mixing the materials^20^.

### Biocompatibility of the bioprinted glioblastoma (GBM) array

**Fig. 4:**
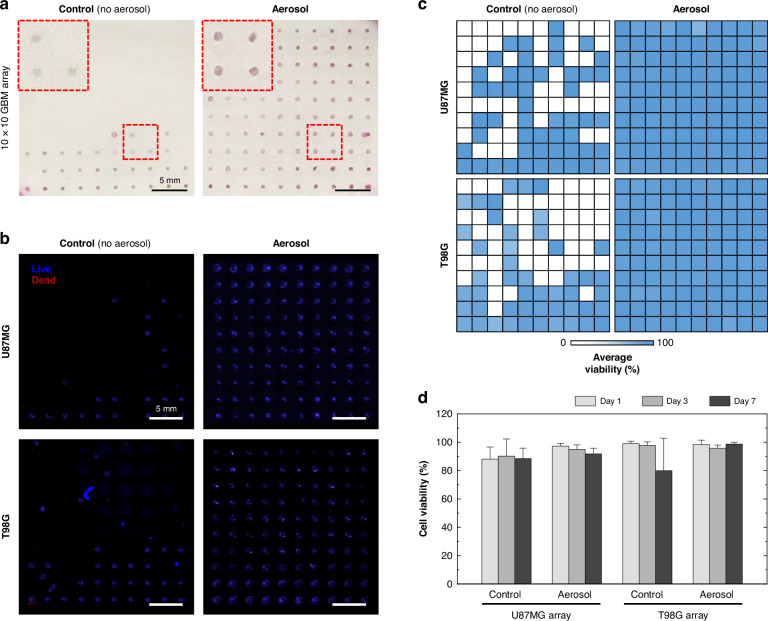
The cell viability test of the bioprinted GBM array. **a** Bright-field images of the 10 × 10 GBM array bioprinted without or with crosslinking aerosol. **b** Live/dead staining images at day 7 of culture in the U87MG and T98G arrays to compare the effect of the crosslinking aerosol during a printing process. **c** A heat map representing the cell viability of each GBM dot of the U87MG and T98G arrays (*n* = 3). **d** Average cell viability of whole arrays consisting of 100 dots over the GBM cell types and crosslinking approaches (*n* = 3)

To recapitulate GBM heterogeneity in the model, two different cell lines of the GBM were selected as the representative glioblastoma cell lines because they have different temozolomide (TMZ) responses. While the U87MG cells are sensitive to TMZ, the T98G cells are resistive to TMZ. Before fabricating a brain tumor model considering the tumor heterogeneity, we investigated the biocompatibility of the bioprinted GBM array consisting of U87MG or T98G bioink. Two distinct 10 × 10 arrays were bioprinted by extruding U87MG or T98G bioinks through a single-barrel nozzle. The GBM array bioprinted under the crosslinking aerosol exhibited more stable adherence of the cell–hydrogel dots on a bottom surface than the control group (Fig. 4a). During seven days of culture, the two types of GBM array were stained with live/dead assay kit on days 1, 3, and 7 to calculate cell viability. Although the hydrogel dots were exposed to fluid flow from the reagent treatment during staining, the GBM array in the aerosol group demonstrated stable adherence on the bottom surface. The nuclei of live and dead cells within 100 hydrogel dots were well stained, exhibiting blue and red fluorescence (Fig. 4b). In contrast, the U87MG and T98G array models in the control group contained fewer GBM dots and showed unstable arrays. The average viability obtained from three array models was visualized as a heat map (Fig. 4c). The control group showed more cell–hydrogel dots detached from the bottom surface and irregular array data of cell viability lower than the aerosol group because of the evaporation issue. In the aerosol group, the 100 dots of U87MG and T98G array showed regular array data and high cell viability. The crosslinking aerosol could not only improve the adherence of the cell-containing hydrogel dots on the bottom surface but also maintain the high cell viability of U87MG and T98G over time (Fig. 4d). We confirmed that the two different GBM arrays bioprinted under the crosslinking aerosol provided biocompatible culture environments.

### Multi-compositional GBM array mimicking intra-tumor heterogeneity

**Fig. 5:**
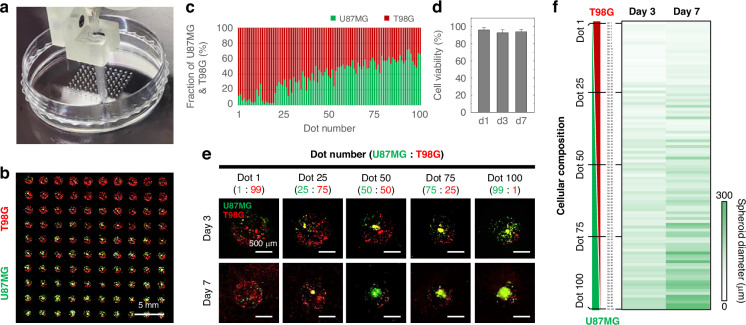
Bioprinted multi-composition GBM array recapitulating intra-tumor heterogeneity in a single in vitro system. **a** Bioprinting of multi-composition 10 × 10 GBM array on a culture dish using the multi-bioink printing system. **b** A bioprinted multi-composition GBM array consisted of green fluorescence-labeled U87MG and red fluorescence-labeled T98G. **c** Fraction of U87MG and T98G contained in each hydrogel dot of the bioprinted GBM array. **d** Average viability of whole arrays consisting of 100 dots at days 1, 3, and 7 of culture (*n* = 3). **e** Morphological changes of U87MG and T98G cultured in each hydrogel dot of the bioprinted multi-composition GBM array. **f** A heat map visualizing the spheroid diameter of 100 GBM dots with different compositions of U87MG and T98G (*n* = 3)

We aimed to fabricate a multi-composition GBM array that reflects intra-tumor heterogeneity, allowing for obtaining various types of data from the single array by providing a tumor microenvironment conducive to cell-to-cell communication. Tumor heterogeneity is a major problem in the treatment of GBM, a brain tumor with a high mortality rate^39–41^. GBM is characterized not only inter-tumor heterogeneity, which represents diversity between patients but also intra-tumor heterogeneity, which represents phenotypical and molecular sub-populations within tumor tissue^42^. Additionally, intercellular communication between different types of cells in intra-tumor heterogeneity of brain cancer is important to maintain the growth and development of GBM cells^39,42,43^. To recreate such an interactive microenvironment reflecting the intra-tumor heterogeneity of brain tumors, we utilized the multi-compositional GBM array. The tumor heterogeneity causes critical problems in treating the brain tumor due to drug resistance^44^. However, there was no in vitro GBM model that could establish the various culture environments and heterogeneous conditions and analyze them at once. Based on the combinational printing technique using two GBM bioinks, a multi-compositional GBM array was printed by combining two bioinks, gelated with a crosslinking solution, and used for cell culture (Fig. S2). 100 GBM dots were sequentially printed with a multibarrel nozzle by gradually adjusting the combination ratio of U87MG and T98G bioinks (Fig. 5a). The multi-composition GBM array bioprinted under the crosslinking aerosol exhibited opposing gradients of U87MG (green fluorescence) and T98G (red fluorescence), as shown in Fig. 5b. The composition of each GBM dot was analyzed by calculating the cellular fraction based on the green and red fluorescence signals (Fig. 5c). The fraction of U87MG and T98G showed a linear gradient, indicating that the cellular composition of the GBM dots varied linearly at each dot. We could confirm that the multi-composition GBM array consisted of 100 different mixing ratios of U87MG and T98G bioinks. The average cell viability calculated from 100 GBM dots was higher than 90% at 1, 3, and 7 days (Fig. 5d). Additionally, each of the 100 GBM dots exhibited cell viability of more than 90% at days 1, 3, and 7 of culture (Fig. S3). On day 3 of culture, we observed U87MG and T98G cells homogeneously distributed within each hydrogel dot (Fig. 5e, f). On day 7 of culture, as the proportion of U87MG cells increased, larger U87MG spheroids formed within the hydrogel. We could observe that controlling various co-culture environments representing intra-tumor heterogeneity of brain tumors resulted in differences in cell growth and development. We confirmed the biocompatibility of the bioprinted GBM array and established the bioprinting technique for the multi-composition GBM array, which can provide 100 different intra-tumor heterogeneity models on the single array.

### Evaluation of temozolomide (TMZ) efficacy by the intra-tumor heterogeneity of GBM

**Fig. 6:**
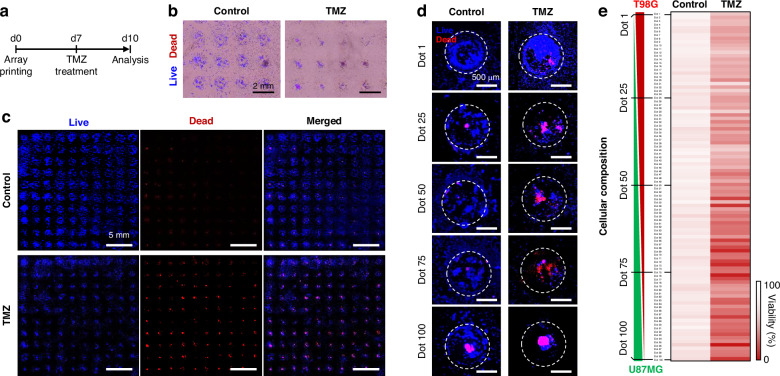
Assessment of TMZ on the multi-composition GBM array representing the intra-tumor heterogeneity. **a** Timeline for bioprinting, TMZ treatment, and analysis. **b** Bright-field images merged with fluorescence images for live and dead cells to observe the distribution and location of the GBM cells. **c** Live/dead staining of the multi-composition GBM array to analyze the TMZ efficacy. **d** Comparison of cellular responses in individual dots of the non-treated and TMZ-treated GBM array. **e** A heat map visualizing the cell viability of 100 GBM dots with different compositions of U87MG and T98G to analyze the TMZ efficacy in diverse co-culture conditions (*n* = 3)

The multi-composition GBM array, representing the intra-tumor heterogeneity, was used to evaluate the efficacy of TMZ. TMZ is commonly used as the first-line chemotherapeutic drug for post-surgical treatment of GBM since the TMZ methylates purine bases of DNA and reduces the cell proliferation rate causing cell death^39^. Although the chemotherapy of TMZ is effective in treating GBM, it has a high recurrence rate due to drug resistance to the heterogeneity of brain tumors^45^. To evaluate TME efficacy by the intra-tumor heterogeneity of GBM, we treated the 790 μM TMZ on the bioprinted GBM array, which has 100 different cellular compositions on day 7 of culture for 3 days (Fig. 6a). The 790 μM TMZ was the median of the IC50 values of T98G and U87MG cells obtained from the single-composition GBM array (Fig. S4). When the T98G or U87MG array was screened with TMZ concentrations ranging from 10 μM to 3 mM, the viability of U87MG decreased more rapidly with increasing TMZ concentration compared to T98G (Fig. S4a). From the drug screening results, we fitted the data points of two GBM cells and calculated the IC50 value of each GBM cell (Fig. S4b). The IC50 values for U87MG and T98G were 232.43 and 1351.44 μM, respectively (Fig. S4c). These results are consistent with previous reports showing that U87MG cells are more sensitive to TMZ than T98G cells^43^, and the IC50 value of the TMZ drug also showed similar values to existing literature^46,47^. Based on the IC50 values of the two GBM cells, the TMZ concentration at which less than half of U87MG died and half of T98G survived was selected as 790 μM and treated to the bioprinted multi-composition GBM array.

After treating the multi-composition GBM array with TMZ for 3 days, we analyzed cellular responses in the intra-tumor heterogeneity model by staining live and dead cells. Compared to the untreated group, the TMZ-treated GBM array exhibited fewer cells around the hydrogel dots (Fig. 6b). These results can be attributed to the effects of TMZ, which is known to inhibit cell growth and migration^45^. Fluorescence images of the entire arrays revealed that the TMZ-treated group exhibited more dead cells in the array (Fig. 6c). In addition, as the dot number of the multi-composition GBM array increased from 1 to 100, the ratio of U98MG cells in the hydrogel dot was gradually higher expressing a stronger fluorescence dead cells’ signal (Fig. 6d). Despite the TMZ treatment, more GBM cells were observed around the hydrogel, attributed to the higher ratio of TMZ-resistive T98G cells. The cell viability in 100 different compositions of the GBM array was visualized as a heat map for intuitive comparison of the control group and TMZ-treated group, as shown in Fig. 6e. The multi-composition GBM array in the untreated group exhibited similar cell viability across all dots. In contrast, the hydrogel in the TMZ-treated group exhibited lower cell viability as the proportion of U87MG cells increased. Dots with a high proportion of T98G cells (Dots 1–50) showed overall cell viability >50%, whereas dots with a higher proportion of U87MG cells (Dots 51–100) exhibited overall cell viability of less than 50%. The tendency of correlation between cellular composition and drug response was quantified using Pearson’s correlation coefficient (Fig. S5). In the control group, the cellular composition showed a low correlation with cell viability, with a value of −0.307. On the other hand, the composition in the TMZ group showed a significant difference in cell viability with a value of −0.696, describing a stronger negative correlation. The multi-composition GBM array could represent the intra-tumor heterogeneity of brain tumors on a single array model. Additionally, the interactive cellular responses, such as migration and viability induced by TMZ treatment in various brain tumor microenvironments, could be analyzed at once from the GBM array.

## Conclusion

Microextrusion bioprinting using multi-bioink printing and aerosol-based crosslinking technologies enabled the stable and versatile fabrication of the small volume and multi-composition hydrogel array. The crosslinking aerosol improved the printing stability of the nanoliter-scale hydrogel dots by preventing the spreading and evaporation of the extruded hydrogels during the prolonged printing process. With the bioprinting system, we significantly increased the production yield of the massive hydrogel dots to approximately 99%. Furthermore, the versatile bioprinting technique has the potential to enhance both the number of model replicates and data throughput. We established the bioprinting technique for a multi-composition array, which can implement heterogeneous conditions in a single array and obtain various types of data. The 100 different compositions, incorporating T98G and U87MG, were implemented in a single array representing the intra-tumor heterogeneity of brain tumors and to assess TMZ efficacy. We anticipate that the microextrusion-based multi-composition array printing technique will provide a novel in vitro system capable of recapitulating intra-tumor heterogeneity for reliable assessment of anti-cancer drug efficacy, while also improving model replicates and data throughput by integrating diverse conditions within the single culture system. Furthermore, if we apply the integration technology of the bioprinted in vitro models with the microfluidic platform^33,48^, we expect that precise control of the cell culture environment and various microfluidic assays will be possible.

## Materials and methods

### Microextrusion printing system setup

A microextrusion printer was prepared to dispense bioinks and print a multi-composition array following our previous setup protocol^22^. In brief, a printhead of a general fused deposition method 3D printer based on the Marilin firmware embedded in a microcontroller (Arduino UNO; Arduino, Italy) was customized to install a printing nozzle and an aerosol tube. For a general hydrogel array, a syringe with a blunt nozzle of 30 gauge was used to extrude a single bioink. When printing a multi-composition array, a syringe nozzle was changed with a multibarrel nozzle which can continuously extrude up to seven bioinks. The multibarrel nozzle, which has an outer diameter of about 400 μm, was prepared through thermal pulling and mechanical grinding of a commercial seven-barrel glass capillary (7B120F-4; World Precision Instruments, FL, USA) and then connected the individual barrel to the pneumatic pumps (MFCS-EZ; Fluigent, France) with Tygon tubes. A commercial nebulizer was modified to deliver the aerosol to a printing bed via a polyvinyl chloride tube (an inner diameter of 9.5 mm and an outer diameter of 12.7 mm). Both the printing nozzle and aerosol tube were held in a printhead of the extrusion bioprinter and the multi-bioink was printed following a programmed coordinate. The multi-bioink printing system was operated in the biological safety cabinet to avoid biological contamination.

### Hydrogel ink, GBM bioinks, and crosslinking agents

As a hydrogel for bioink, the 2% (w/v) alginate-RGD (NOVATACH MVG GRGDSP; Novamatrix, Sandvika, Norway) was prepared by diluting with phosphate-buffered saline (PBS). 10% (w/v) and 2% (w/v) CaCl_2_ (CaCl_2_ dihydrate; Sigma, Saint Louis, MO, USA) solution diluted in DW were prepared for primary (aerosol type) and secondary (solution type) crosslinking of the extruded alginate-based ink, respectively. The alginate ink and CaCl_2_ solution were used to verify the effect of the aerosol-based crosslinking approach to gelate alginate dot. To prepare the two different GBM inks, U87MG (1 × 10^6^ cells/mL) or T98G (1 × 10^6^ cells/mL) were mixed in the 2% (w/v) alginate-RGD. 10% (w/v) CaCl_2_ was used as a crosslinking aerosol while the GBM inks were printed. Before all experiments, the hydrogel precursors and crosslinking solution were sterilized with a 0.2 μm filter to avoid contamination.

### Analysis of the morphology of the hydrogel dot by the crosslinking aerosol effect

A printing substrate was prepared by bonding a PDMS frame on a 2 × 3 inch glass slide-through plasma treatment to print hydrogel dots. After plasma bonding, the substrate was sonicated with 70% ethanol, rinsed with deionized water, and dried in an oven. The alginate ink was extruded on a glass slide through a syringe nozzle and exposed to the external environment without or with 10% (w/v) CaCl_2_ aerosol. For 400 s after the hydrogel dot was extruded, the morphological change of the dot was analyzed with the side view images over time. The time-lapsed images of the hydrogel dots were obtained from the contact angle analyzer (Phoenix-MT; SEO Co., Ltd, Suwon, Korea) to compare the effect of the crosslinking aerosol. To analyze the evaporation and spreading of the extruded hydrogel ink, dot volume and contact angle were measured from the side view images for 400 s on software provided by the manufacturer.

### Fabrication of nanoliter-scale hydrogel arrays with improved printing resolution and stability

To compare the effect of the crosslinking aerosol and analyze technical issues that occurred during the printing process, the 10 × 10 hydrogel array consisting of 100 dots, which has sub-hundreds nanoliters, was printed through a syringe nozzle of 30 gauge (inner diameter of 140 μm and outer diameter of 310 μm) installed in the microextrusion bioprinting system for 400 s. In our experimental setup, the aerosol could not only gelate the extruded bioink on the substrate but also the alginate around the printing nozzle. To avoid the gelation of the alginate ink around the printing nozzle and to keep the ink on the substrate, a suitable volume of the ink was extruded, adjusting the printing conditions. When the alginate ink was printed at a printing speed of 100 cm/min under a pneumatic pressure of 350 mbar, the printing conditions allowed for stable printing of the alginate array on the substrate while minimizing gelation around the nozzle. The printed hydrogel dots were categorized into three states (moist, evaporating, and evaporated) based on the image analysis of the individual dots depending on the boundary of the hydrogel dots. From the image analysis, the array compositions in the control and aerosol groups were compared with the ratio of the hydrogel dots with different states. The dot volume of the 10 × 10 array was measured from the image obtained in the contact angle analyzer. As the hydrogel dots evaporated quickly after printing, the array samples were immediately moved to the contact angle analyzer. The images were then captured rapidly, minimizing the volume loss by evaporation. To investigate the stability of the printed array, the dot attachment ratio was calculated by counting the adhered hydrogel dots on the substrate after adding the solution to the printed array. With the stable array printing technique, the feasibility of mass production of hydrogel dots to provide the improved model replicates was demonstrated by extruding the nanoliter scale hydrogel array consisting of 600 hydrogel dots at a speed of 1.5 dots/s.

### Analysis of the multi-composition hydrogel array with various patterns

The multi-bioink printing system was demonstrated to fabricate multi-composition hydrogel arrays with several alginate inks, including fluorescence beads, through the multibarrel nozzle under the crosslinking aerosol. The row-by-row patterned hydrogel array was printed with three alginate inks by switching the pneumatic pressure of 350 mbar to change the inks. With the combination printing technique, the hydrogel array with 100 different compositions was printed with only two alginate inks by adjusting the ratio of the two inks with the pneumatic pumps. Two alginate inks, including red and green fluorescence beads, were printed by applying pneumatic pressure with a polynomial profile from 50 to 350 mbar. When printing the multi-composition array via switching three bioinks or combining two bioinks, the pneumatic pumps were controlled following a command programmed on LabVIEW software (LabVIEW; National Instruments, Austin, TX, USA). The composition of the printed array was analyzed with the ratio of the RGB values of the fluorescence images obtained in each dot.

### GBM cell maintenance

The U87MG and T98G cells were purchased in the Korean Cell Line Bank and cultured in Dulbecco’s modified Eagle Medium (DMEM) containing 10% fetal bovine serum and 1% penicillin–streptomycin. The DMEM culture medium was exchanged every two or three days. When printing the GBM arrays, the two types of GBM cells were used as bioinks by mixing with alginate-RGD after detaching.

### Bioprinting of single and multi-composition GBM arrays

The single and multi-composition GBM arrays were prepared following the printing procedure of Fig. S2. The single-composition array with T98G or U87MG cells was printed on the culture dish and used to verify the biocompatibility of the bioprinting process with the 10% (w/v) CaCl_2_ aerosol. The multi-composition GBM array was prepared to analyze cell growth and drug responses in the microenvironment considering intra-tumor heterogeneity using the microextrusion-based multi-bioink printing system. After the bioprinting process of the single or multi-composition GBM array, the array was submerged in the 2% (w/v) CaCl_2_ solution to fully crosslink the alginate for 5 min. The GBM array was washed with PBS, and the cells were cultured in DMEM. The culture medium was exchanged every two days. When printing the multi-composition GBM array, U87MG and T98G cells were identified by staining with CellTracker of green CMFDA dye (C7025; Thermo Fisher, Norcross, GA, USA) and red CMTPX dye (C34552; Thermo Fisher) according to the protocol of the manufacturer, respectively.

### Cell viability assay

Single- and multi-composition GBM arrays were stained with live/dead assay kits (ReadyProbes™ cell viability imaging kit, blue/far-red; Invitrogen, Waltham, MA, USA). The live and dead staining solution was prepared by mixing with the DMEM. The GBM array was immersed in the staining solution in a 37 °C CO_2_ incubator for 30 min following the manufacturer protocol. The live and dead cells were observed at specific wavelengths on a digital fluorescence microscope (F1-CIS; Nanoscope Systems, Daejeon, Korea). The fluorescence images of 100 dots in the GBM array were individually analyzed to visualize and compare the cellular responses. The fluorescence signals of each image were measured on ImageJ software (https://imagej.nih.gov/ij/), and cell viability was calculated as a ratio of the area of the total cells’ nuclei (blue fluorescence) and dead cells’ nuclei (far-red fluorescence). Before the drug test on the multi-composition GBM array, a range of TMZ concentrations from 10 μM to 3 mM on the single-composition GBM array with U87MG or T98G cells was screened to determine suitable concentration based on the IC50 value. The IC50 value was calculated by fitting a curve on a drug screening plot. The 790 μM TMZ, which was the mean between IC50 values of U87MG and T98G arrays, was treated on the multi-composition GBM array to investigate the drug efficacy in the microenvironment considering intra-tumor heterogeneity. The viability value of the GBM cells cultured in each hydrogel dot in the printed array was visualized with a heat map.

### Preparation and treatment of TMZ

TMZ (5.00609; Sigma) stock solution was prepared by dissolving with DMSO following a manufacturer protocol and stored at −20 °C freezer. After a thawing process of the TMZ stock, the TMZ solution was diluted with DMEM growth medium to prepare various concentrations. To conduct a drug test, the TMZ-containing DMEM was treated on single- or multi-composition GBM arrays at day 7 of culture. After TMZ treatment for 3 days, the GBM array was washed with PBS and the cellular responses were analyzed.

### Morphological analysis of the GBM spheroids

To recognize the U87MG in the co-culture environment, the U87MG cells were stained with CellTracker of green CMFDA dye. The green fluorescence-labeled U87MG cells were captured on a microscope at days 3 and 7 of culture to analyze the cellular morphology within the hydrogel dots over time. The fluorescence images were obtained, exhibiting morphological changes of the U87MG from single cells to the formation of spheroids within the hydrogel. The green fluorescence area representing the size of the U87MG spheroid was measured from the microscopic images of each hydrogel dot on the ImageJ software.

### Statistical analysis

The data in bar plots represented the mean ± standard deviation. After performing the normality test, statistical significance was analyzed using paired *t*-tests on IBM SPSS Statistics (IBM, Armonk, NY, USA). The correlation between cellular composition and drug response with control and TMZ groups was quantified with Pearson’s correlation coefficient. The statistical significance was represented with a *p*-value of <0.001 (***).

## Supplementary information

Supplementary information (Figures S1~S5)
