# Supplementary material for: Bioprinting of a multi-composition array to mimic intra-tumor heterogeneity of glioblastoma for drug evaluation
Source: Microsyst Nanoeng. 2024 Dec 11;10:186. doi: 10.1038/s41378-024-00843-w (PMC11634888; doi:10.1038/s41378-024-00843-w)
Supplement: Supplementary file 1 — Supplementary information (Figures S1~S5) [file 41378_2024_843_MOESM1_ESM.pdf]

Supplementary information

Bioprinting of a multi-composition array to mimic intra-tumor heterogeneity of glioblastoma for drug evaluation

Gihyun Lee[1], Soo Jee Kim[1], Yejin Choi[1], Jongho Park[1] and Je-Kyun Park*[,1,2,3]

[1] Department of Bio and Brain Engineering, Korea Advanced Institute of Science and Technology (KAIST), 291 Daehak-ro, Yuseong-gu, Daejeon 34141, Republic of Korea.

[2] KI for Health Science and Technology, KAIST Institutes (KI), 291 Daehak-ro, Yuseong-gu, Daejeon 34141, Republic of Korea

[3] KI for NanoCentury, KAIST Institutes (KI), 291 Daehak-ro, Yuseong-gu, Daejeon 34141, Republic of Korea

Correspondence: Je-Kyun Park (jekyun@kaist.ac.kr)

Fig. S1. Analysis of the hydrogel dots of the 10 × 10 array bioprinted without or with the crosslinking aerosol.

Fig. S2. Preparation steps for the 10 × 10 GBM array with multibarrel nozzle and the crosslinking aerosol.

Fig. S3. A heat map visualizing the cell viability of 100 GBM dots with different compositions of U87MG and T98G at days 1, 3, and 7 of culture (*n* = 3).

Fig. S4. TME screening to calculate IC50.

Fig. S5. Pearson's correlation coefficient between the cellular composition and viability in the multi-composition GBM array in the control and TMZ groups.

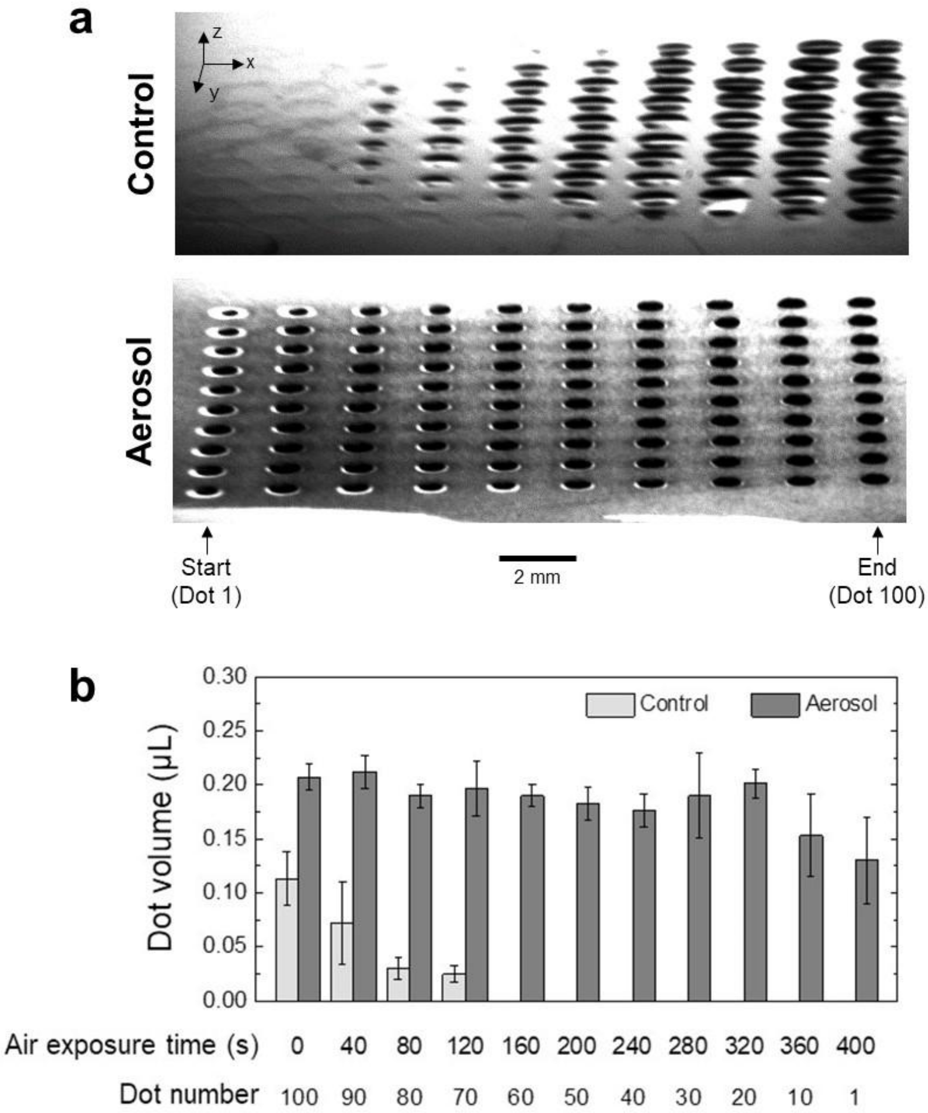

Fig. S1. Analysis of the hydrogel dots of the 10 × 10 array bioprinted without or with the crosslinking aerosol. a A bird's eye view of the 10 × 10 array captured with a contact angle analyzer. **b** Hydrogel dot volume of each dot of the array ($n = 4$).

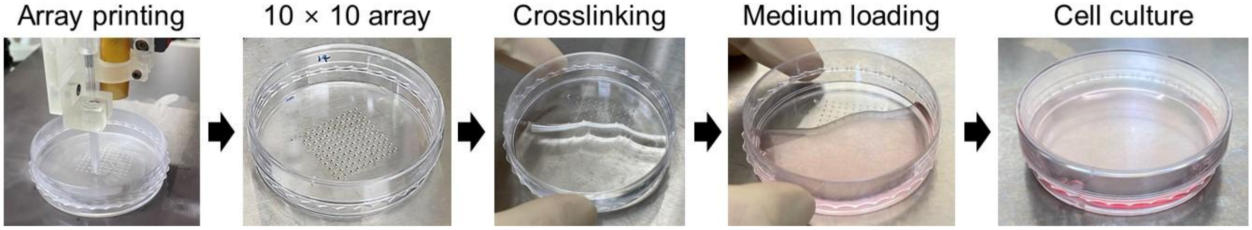

Fig. S2. Preparation steps for the 10 × 10 GBM array with multibarrel nozzle and the crosslinking aerosol.

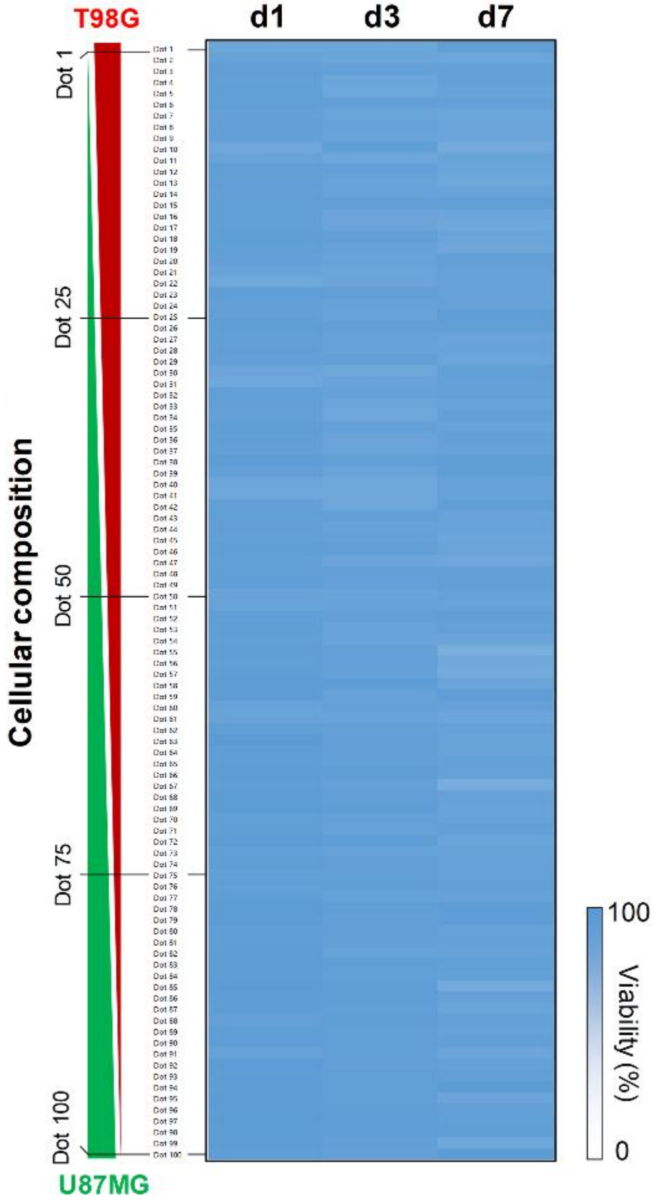

Fig. S3. A heat map visualizing the cell viability of 100 GBM dots with different compositions of U87MG and T98G at days 1, 3, and 7 of culture (*n* = 3).

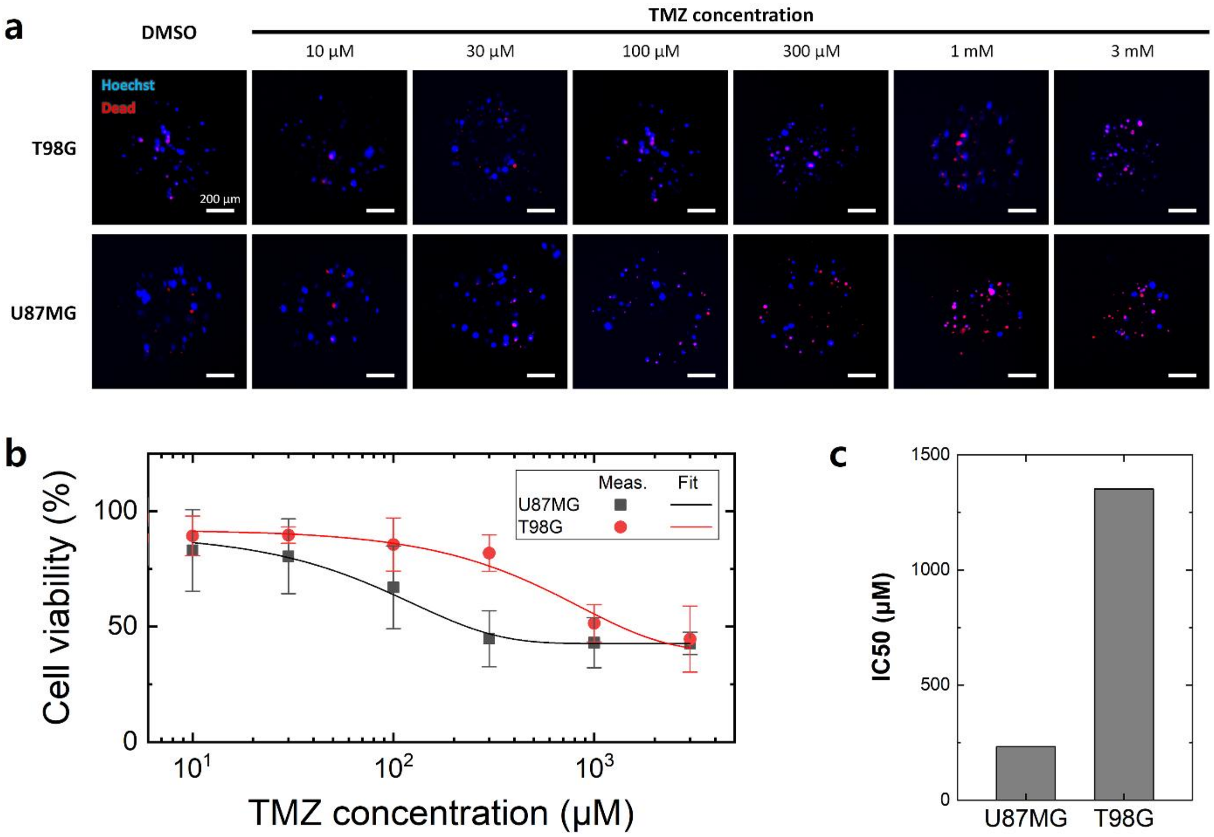

Fig. S4. TME screening to calculate IC50. **a** Live/dead staining images of the individual T98G and U87MG dots after screening with TMZ from 10 μM to 3 mM for 3 days (n = 3). **b** TMZ screening profile of T98G and U87MG arrays with fitting lines (n = 3). **c** IC50 values of U87MG and T98G calculated from the TMZ screening plot.

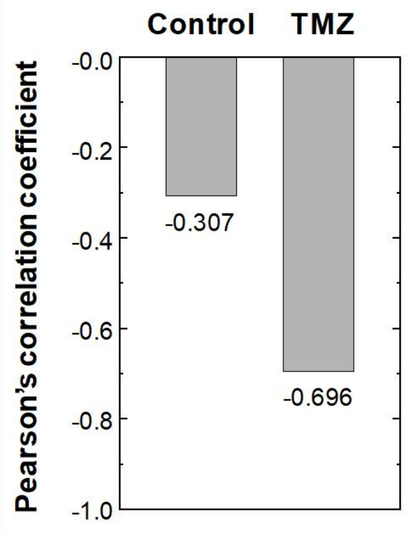

Fig. S5. Pearson's correlation coefficient between the cellular composition and viability in the multi-composition GBM array in the control and TMZ groups.
